# Towards microalgal triglycerides in the commodity markets

**DOI:** 10.1186/s13068-017-0873-2

**Published:** 2017-07-17

**Authors:** Giulia Benvenuti, Jesús Ruiz, Packo P. Lamers, Rouke Bosma, René H. Wijffels, Maria J. Barbosa

**Affiliations:** 1 Bioprocess Engineering, AlgaePARC, Wageningen University, P.O. Box 16, 6700 AA Wageningen, The Netherlands; 2 Algades–Alga, Development, Engineering and Services, S.L., c. Margaritas, Costa Oeste, El Puerto de Santa María, 11500 Cádiz, Spain; 3 Biosciences and Aquaculture, Nord University, 8049 Bodø, Norway

**Keywords:** Microalgae, Triglyceride production, Techno-economic analysis, Production costs

## Abstract

**Background:**

Microalgal triglycerides (TAGs) hold great promise as sustainable feedstock for commodity industries. However, to determine research priorities and support business decisions, solid techno-economic studies are essential. Here, we present a techno-economic analysis of two-step TAG production (growth reactors are operated in continuous mode such that multiple batch-operated stress reactors are inoculated and harvested sequentially) for a 100-ha plant in southern Spain using vertically stacked tubular photobioreactors. The base case is established with outdoor pilot-scale data and based on current process technology.

**Results:**

For the base case, production costs of 6.7 € per kg of biomass containing 24% TAG (w/w) were found. Several scenarios with reduced production costs were then presented based on the latest biological and technological advances. For instance, much effort should focus on increasing the photosynthetic efficiency during the stress and growth phases, as this is the most influential parameter on production costs (30 and 14% cost reduction from base case). Next, biological and technological solutions should be implemented for a reduction in cooling requirements (10 and 4.5% cost reduction from base case when active cooling is avoided and cooling setpoint is increased, respectively). When implementing all the suggested improvements, production costs can be decreased to 3.3 € per kg of biomass containing 60% TAG (w/w) within the next 8 years.

**Conclusions:**

With our techno-economic analysis, we indicated a roadmap for a substantial cost reduction. However, microalgal TAGs are not yet cost efficient when compared to their present market value. Cost-competiveness strictly relies on the valorization of the whole biomass components and on cheaper PBR designs (e.g. plastic film flat panels). In particular, further research should focus on the development and commercialization of PBRs where active cooling is avoided and stable operating temperatures are maintained by the water basin in which the reactor is placed.

**Electronic supplementary material:**

The online version of this article (doi:10.1186/s13068-017-0873-2) contains supplementary material, which is available to authorized users.

## Background

Currently, microalgal products are mainly sold in niche markets [1]. Commercially relevant microalgal products are basically biomass or extracts rich in PUFAs (EPA, DHA), essential amino acids and antioxidants (carotenoids, tocopherols and phenols) as supplements for human health and cosmetics [2, 3]. However, in the last 10 years, industry has been looking at alternative and sustainable feedstocks for commodities in the food, feed, chemical and biofuel sectors. This is mainly due to the social and political awareness for sustainability, the instability of fossil fuel prices, the pressure on agriculture crops for non-food applications, the growth in population and limited availability of arable land. In this context, microalgal triglycerides (TAGs) are regarded as an attractive source to supplement or substitute oils derived from fossil resources and/or agricultural crops [4, 5]. Microalgae can grow on non-arable land and they have a low freshwater and fertilizer footprint when grown on wastewater, sea- or brackish water [4]. Besides TAGs, other valuable products are obtained [6], improving the range of marketable products and consequently the commercial feasibility. A commercial microalgal bulk industry would represent an enormous incentive for national economies. Malik et al. [7] calculated that the production of 1 Mton of bio-crude oil from microalgae could generate 13,000 new jobs in Australia and turnover of 2.6 billion €. Despite the high potential of microalgae as sustainable TAG cell factories, microalgal TAGs are not yet commercialized, mainly due to process immaturity and estimated high production costs [8].

To determine research priorities and support business decisions, solid techno-economic studies are essential. Although several studies have been published on N-replete biomass [8–10], only a few specifically focus on processes targeted to the production of TAG-enriched biomass under so-called “stress” conditions (e.g. N-starvation).

Here, we present projection on a two-step 100-ha-scale TAG production process in which, in the first step, biomass is grown under nitrogen replete conditions in continuously operated photobioreactors (“growth” PBRs) and, in the second step, multiple batch-operated stress (i.e. nitrogen starvation) PBRs are inoculated and sequentially harvested, thus ensuring a daily harvest of TAG-enriched biomass. Noteworthy, in our study, photosynthetic efficiencies obtained in outdoor pilot cultivations of *Nannochloropsis* sp. CCAP 211/78 [11, 12] are used as model input to conduct the techno-economic analysis for the mentioned production facility. Furthermore, our study considers location-specific parameters such as climate, labour and energy costs, which highly affect productivity and economic profitability. The production costs of TAG-enriched biomass are presented based on current process technology. Finally, a sensitivity analysis is performed and scenarios with reduced production costs are discussed in the light of the latest biological and technological advances, indicating the roadmap towards a further cost reduction.

## Methods

In the following section, a techno-economic analysis of a two-step TAG production is presented and used as basis to indicate guidelines for future research. The solid techno-economic model originally developed by Ruiz et al. [13] for nitrogen replete biomass production was extended with the TAG production phase. Our evaluation includes the cultivation phase in “growth” and “stress” PBRs and the biomass concentration step to obtain 15% w/w algal slurry as final product. The production costs of TAG-enriched biomass are presented.

In our process, nitrogen (N) replete biomass is produced in continuous (chemostat)-operated PBRs (“growth PBRs”) to ensure a continuous supply of inoculum for the TAG accumulation phase for which multiple batch-operated “stress PBRs” are sequentially inoculated and harvested at maximum time-averaged TAG productivity [14] ensuring a constant daily harvest of TAG-enriched biomass (Additional file 1: Fig. A1). Projections were made for a 100-ha-scale plant using vertically stacked tubular PBRs in southern Spain (37°15′ N 6° 56′ W). The design of the PBRs is identical to the vertically stacked horizontal tubular photobioreactors described by [13].

### Process description

**Fig. 1:**
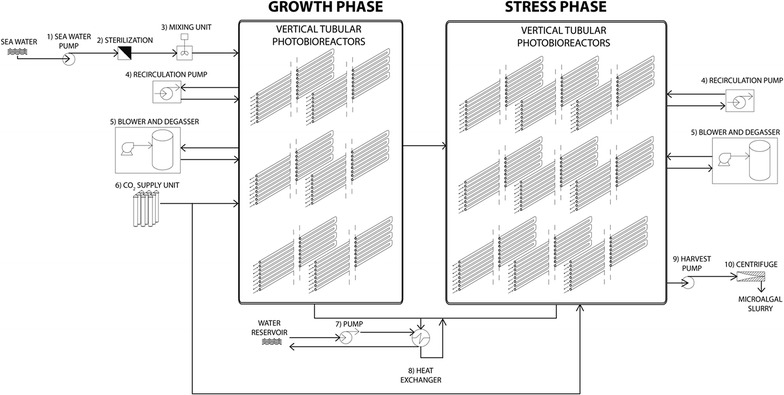
Plant configuration. Schematic representation of the plant as adopted for the base case projection (see “Process description”)

The production area (100 ha) is divided into two stages: the growth phase, where biomass is produced in chemostat-operated PBRs, and the stress phase, producing TAG-enriched biomass in batch-operated PBRs under nitrogen starvation (Fig. 1).

The process starts by filtering natural seawater from the surface, which is then mixed with nutrients in an automatized mixing unit and pumped into the growth PBRs. The seawater-based medium enters the growth PBRs only during daylight hours, and, concurrently, the same culture volume leaves the reactors. This outflow sequentially fills different stress PBRs to which no nutrients are added to promote TAG accumulation. Culture medium is enriched with different nutrients [12] but only the cost of nitrogen, phosphorus and CO_2_ is considered being those with major influence on economics [13].

In each stress PBR unit, the TAG accumulation phase starts immediately after inoculation. For this, it is assumed that the culture leaving the growth PBRs is free of extracellular nitrogen source with no impact on the biomass productivity. In practice, a near-zero extracellular nitrogen concentration can be achieved by adopting nitrogen sensors, turbidity sensors (using an empirically determined nitrogen content of biomass) or off-gas-based dosing (using empirical stoichiometric relations).

In the stress phase, independent PBR units are harvested sequentially, resulting in a batch strategy with a certain retention time. Thus, from the entire stress area there is a constant daily harvest of TAG-enriched biomass. When the stress PBRs are harvested, the TAG-enriched biomass is pumped into the centrifuge where 15% algal slurry is obtained. In both growth and stress PBRs, the culture is mixed by a recirculation pump at a horizontal liquid velocity in the tubes of a flow of 0.45 m s^−1^, typically used in this type of photobioreactor [11]. The culture is supplied with pure CO_2_ on demand to keep pH at the set point of 7.5 [12]. To prevent oxygen inhibition, degassers ensure that oxygen partial pressure never exceeds 300% [15], equivalent to three times saturation with respect to air.

Culture temperature is maintained below 30 °C with heat exchangers that recirculate seawater from a depth of 200 m. Wastewater treatment is not performed because the effluent of the stress PBRs is considered free of nutrients and organic matter. The plant is operational for 300 days per year, three cleanings per year are performed and one plant manager, three supervisors and 28 operators are required to run the facility [13].

### Empirical data and area allocation

For the base case, yearly biomass and TAG productivities were calculated using the photosynthetic efficiencies (i.e. fraction of total light energy converted into chemical energy during photosynthesis) obtained outdoors at AlgaePARC pilot facility in the Netherlands ([11] for biomass production and [12] for TAG production) and the total irradiance in southern Spain [13] (Additional file 1: Eq. A1). For the growth phase, an average photosynthetic efficiency of 2.17% for biomass production (containing 4% TAG w/w) and a daily culture dilution rate of 27% were used [11]. This means 3.7 days of retention time of culture in growth phase. For the stress phase, an average photosynthetic efficiency of 1.48% for biomass production (containing 24% TAG w/w) was used (Additional file 1: Table A1; [12]). Based on the data obtained at pilot scale in the Netherlands [11, 12], we calculated that under low-light conditions in the Netherlands (14 mol m^−2^ d^−1^), total TAG productivity is maximal after 9 days in the stress reactor, whereas at high-light conditions (36 mol m^−2^ d^−1^), the productivity is maximal after 6 days. Because southern Spain has longer periods of high light compared to the Netherlands, we chose to always harvest the TAG-enriched biomass after 6 days in the stress PBRs. This retention time of 6 days can thus be regarded as a 17% daily dilution of the PBRs in the stress area.

The total production area (100 ha) was allocated between growth and stress phase using mass balances based on total area and the aforementioned dilution rates. It resulted in areas of 38.2 and 61.8 ha and volumes of 21,014 and 33,700 m^3^ for the growth and stress phases, respectively. 10% of the growth area is allocated to inoculum production to fill the growth PBRs after a routine cleaning or culture crash. The area for inoculum production is considered identical to the growth area in terms of operational and capital costs (OPEX and CAPEX). However, since this biomass is only incidentally transferred to the growth PBRs, the inoculum production area is assumed as non-productive. The area occupied by side equipment and piping is considered as 20% of the production area, thus resulting in a total facility area of 120 ha.

As described by Ruiz et al. [13], the model uses location-specific parameters such as climatic conditions, energy costs (Additional file 1), labour costs and employer’s contribution to labour costs as well as workweek hours (Additional file 1: Table A5). In Additional file 1: Table A2, the changes in major equipment (numbers 1–11 in Fig. 1), capacity and power requirement are reported. These modifications were made due to the different process strategy adopted in this study (i.e. different area, flows and volumes) compared to Ruiz et al. [13]. Furthermore, in Additional file 1: Table A3, the procedure for calculating CAPEX and OPEX is reported. An account of the main model features is given in Additional file 1. For further details, we refer to [13].

## Results

### Production cost of TAG-enriched biomass: base case based on pilot plant data

The photosynthetic efficiencies and TAG contents obtained at pilot scale using current process technology and design were used as model base case (Table 1). A TAG-enriched biomass production cost of 6.7 € kg^−1^ was obtained and the net energy ratio (energy produced as dry microalgae biomass/energy consumed as electricity) was 1.1. The latter indicates that the amount of energy (i.e. chemical energy; Additional file 1: Table A1) generated by the process was slightly higher than the energy required for operating the plant to obtain the algal slurry.

**Table 1:** Results of the techno-economic analysis

	Base case Growth phase: 2.17% PE, 4% TAG w/w Stress phase: 1.48% PE, 24% TAG w/w 30 °C max. culture temperature for cooling Current process technology	Optimized case (1) in Fig. 4) Growth phase: 6% PE, 4% TAG w/w Stress phase: 4.10% PE, 60% TAG w/w 40 °C max. culture temperature for cooling Optimized process technology*
Total costs (M€ year^−1^)	25.6	28.2
Biomass production (Kton year^−1^)	3.8	8.7
TAG production (Kton year^−1^)	0.5	3.7
Biomass cost (€ kg^−1^)	*6.7*	*3.3*
CAPEX (M€ year^−1^)	9.7	14.4
OPEX (M€ year^−1^)	16.0	13.9
Initial investment (M€)	140.8	210.1
Produced energy (GWh year^−1^)	26.0	70.9
Consumed energy (GWh year^−1^)	22.8	38.0
Net energy ratio	1.1	1.9

* Flow velocity is reduced from 0.45 to 0.3 m s^−1^ during the day and to 0.23 m s^−1^ during the night. Harvest is performed by pre-concentration of the biomass by microfiltration followed by centrifugation instead of using centrifugation only; flue gas is used as CO_2_ source instead of commercial CO_2_; 310 operational days per year instead of 300; reduced number of employees (one manager, one supervisor, eight operators instead of one manager, three supervisors and 28 operators); cleaning reduced from three times to one per year; the fraction of the facility used to prepare inoculum is reduced from 10 to 5% of the growth area

**Fig. 2:**
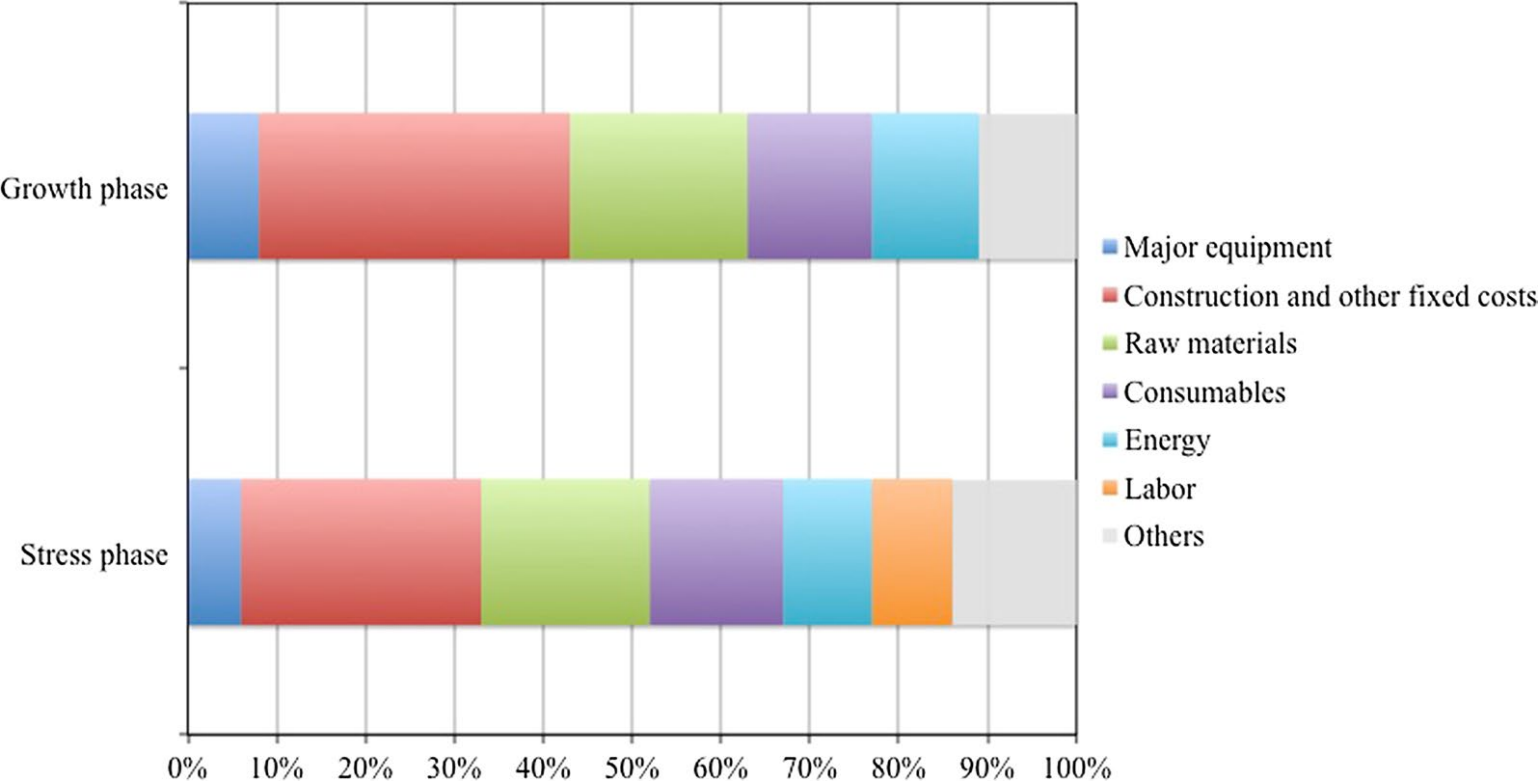
Cost breakdown. Cost breakdown for growth (**a**) and stress (**b**) phases for a two-step-continuous TAG production process in vertically stacked tubular PBRs. Labour costs of the complete facility (100 ha) are assigned to the stress phase to simplify calculations

A cost breakdown analysis was conducted (Fig. 2). Our analysis shows that, in both phases, the largest contribution to total costs (35% for growth and 27% for stress) is given by construction and other fixed costs (i.e. land improvement, installation costs, service facilities, instrumentation and control, electrical, piping, buildings, construction expenses, engineering and supervision, contractor’s fee, contingency), followed by raw materials (i.e. nutrients as well as chemicals and granulate used for cleaning) and consumables (i.e. glass tubes).

**Fig. 3:**
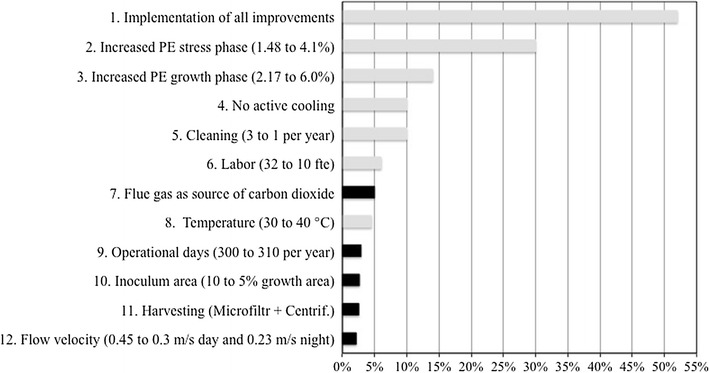
Sensitivity analysis. Effect of individual parameters on cost reduction for TAG-enriched biomass production. Parameters are changed to the values used for future projections *Black bars* refer to short-term improvements; *grey bars* refer to mid-term improvements (see “Opportunities for cost reduction”)

Despite the fact that the largest contribution to total costs (35% for growth and 27% for stress) is given by construction and other fixed costs (Fig. 2, Additional file 1: Table A3), these have not been considered in our sensitivity analysis (Fig. 3). In fact, due to the lack of large facilities (>5 ha) and the novelty of the process, a certain level of uncertainty is related to these costs. We, however, believe that with a more mature technology, construction and fixed costs can be decreased by, for instance, reducing contingencies and the capital required for instrumentation and control.

### Opportunities for cost reduction

To estimate the cost reduction that expected advances in the field could yield, we performed a sensitivity analysis (Fig. 3). For a substantial cost reduction, both biological performance of the production strain and process technology should be optimized. For this, the impact of expected improvements on the production cost was investigated. The individual effect was analysed in isolation to identify key parameters. It is assumed that a single parameter change does not alter the rest of variables. Likewise, it is also presented the case where all these factors are simultaneously changed to the future value (“*Implementation of all improvements*” in Fig. 3 and “Optimized case” in Table 1).”

We consider some of the below-mentioned improvements that are feasible in the short term (within the next 3 years from now) and some in the mid-term (within the next 8 from now).

#### Short-term improvements

- The flow velocity is reduced from 0.45 to 0.3 m s^−1^ during the day [16] and to 0.23 m s^−1^ during the night (with no negative effects on productivity) [17].
- Flue gas instead of commercial CO_2_ is used (with no negative effects on productivity) [18].
- The plant is operational for 310 days per year instead of 300.
- The fraction of the facility used to prepare inoculum is reduced from 10 to 5% of the growth area.
- TAG-enriched biomass is pre-concentrated by microfiltration and subsequently centrifuged. The biomass is concentrated 15 times by membrane filtration at a flow of 32 L m^−2^ h^−1^ [19]. The retentate is then further processed by centrifugation.

#### Mid-term improvements

- An increased photosynthetic efficiency (PE) has been suggested to be one of the major targets to achieve a substantial cost reduction [20, 21]. During the growth phase, the PE is increased to 6% [13, 22] while, for stress phase, the PE is increased proportionally to 4.1%, and the TAG content is augmented to 60% w/w [23]. In such a way, the combined effect of biological and process improvements on TAG productivity, as thoroughly discussed in “How to increase TAG productivity”, is represented.
- The maximum culture temperature at which cooling is started is increased from 30 to 40 °C. Lower cooling requirements could be achieved by selecting or improving strains that can be cultivated with the same productivity at higher culture temperatures [24, 25].
- Active culture cooling can be avoided in floating/submerged cultivation systems (e.g. Algae Floating Systems, Inc., Algasol Renewables, OMEGA PBRs) placed in water bodies close to land or in shallow (artificial) basins in which seawater can be introduced and released based on tide differences.
- Reactors are cleaned once per year instead of three, as a result of a better fouling management and robust process less prone to contamination.
- The number of employees is reduced to one manager, one supervisor and eight operators [9]. For this, high degree of automation and a more mature technology are necessary.

For future projections, the basis of the calculations was identical to the base case with the exception of scenarios (1) and (3) of Fig. 3. For these two scenarios, the photosynthetic efficiency during the growth phase was increased, while the biomass concentration was kept at the same values as in the base case. As a result, a greater dilution rate of 78.5% was used (i.e. hydraulic retention time of 1.27 days). As the flow leaving the growth area must be identical to the flow entering the stress area, the areas and volumes of both growth and stress phases were changed resulting in 17.5 and 82.5 ha and 10,052 and 45,754 m^3^, respectively.

**Fig. 4:**
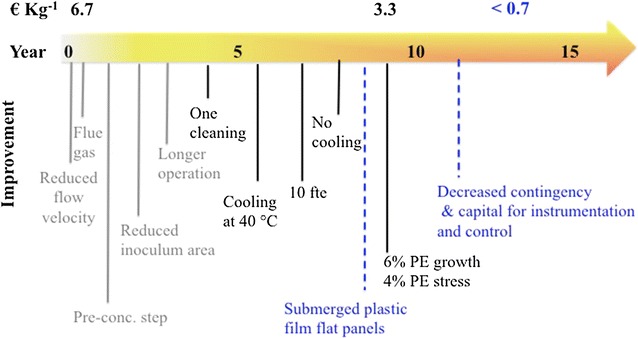
Roadmap towards economically feasible microalgal TAG. Suggested improvements and expected timeframe for their realization are shown. The results of our estimations are reported in *grey* and *black lines*/*font*. The parameters that we expect contributing to further cost reduction are indicated with *blue-dashed lines*/*font*. We expect that the combined effect of all parameters will result in a production cost below 0.7 € kg^−1^ within the next 15 years

With our sensitivity analysis (Fig. 3) we define a roadmap (Fig. 4) for research on microalgae TAG production. Much effort should be focused on increasing the photosynthetic efficiency (i.e. productivity) during the stress and growth phases, as this is the most influential parameter on production costs (30 and 14% cost reduction from base case) [26–28]. Next, biological and technological solutions should be implemented for a reduction in cooling requirements (10 and 4.5% cost reduction from base case when active cooling is avoided and cooling setpoint is 40°C, respectively) (Fig. 3). The development of robust processes, which are less prone to contamination and fouling, thus requiring less cleaning, can save up to 10% compared to the base case. Similarly, reduction of labour costs can be achieved by investing on automation, thus contributing to a 6% reduction in total production costs. If all suggested improvements are combined, TAG-enriched biomass production costs will be substantially reduced from 6.7 to 3.3 € kg^−1^ (with 60% w/w TAG content; Table 1).

Nevertheless, to enter the commodity markets, production costs should decrease even further. Production costs should be at least lower than the selling price (0.7–3.5 € kg^−1^; [13]; Fig. 4). However, without a detailed market analysis, a production cost below market value is not enough to establish economic decisions. We believe that further cost reduction can be achieved when contingencies will be decreased due to a more mature technology, as well as the capital for instrumentation and control. Next, plastic film flat panels, instead of tubular systems, should be used as they are more productive, require lower installation and operational costs and produce a culture with a greater biomass concentration [13]. Active cooling should be avoided. For this, potential relies on floating/submerged cultivation systems. By joining forces in these research areas, we believe that production cost below 0.7 € kg^−1^ can be achieved within the next 15 years (Fig. 4).

To ensure economic viability of microalgal TAGs at the current commodity market values, the other biomass components should be valorized as well as the selling price of microalgal TAGs should be increased (e.g. enriching the TAG composition in specific fatty acids with higher market price).

## Discussion

### How to increase TAG productivity

**Fig. 5:**
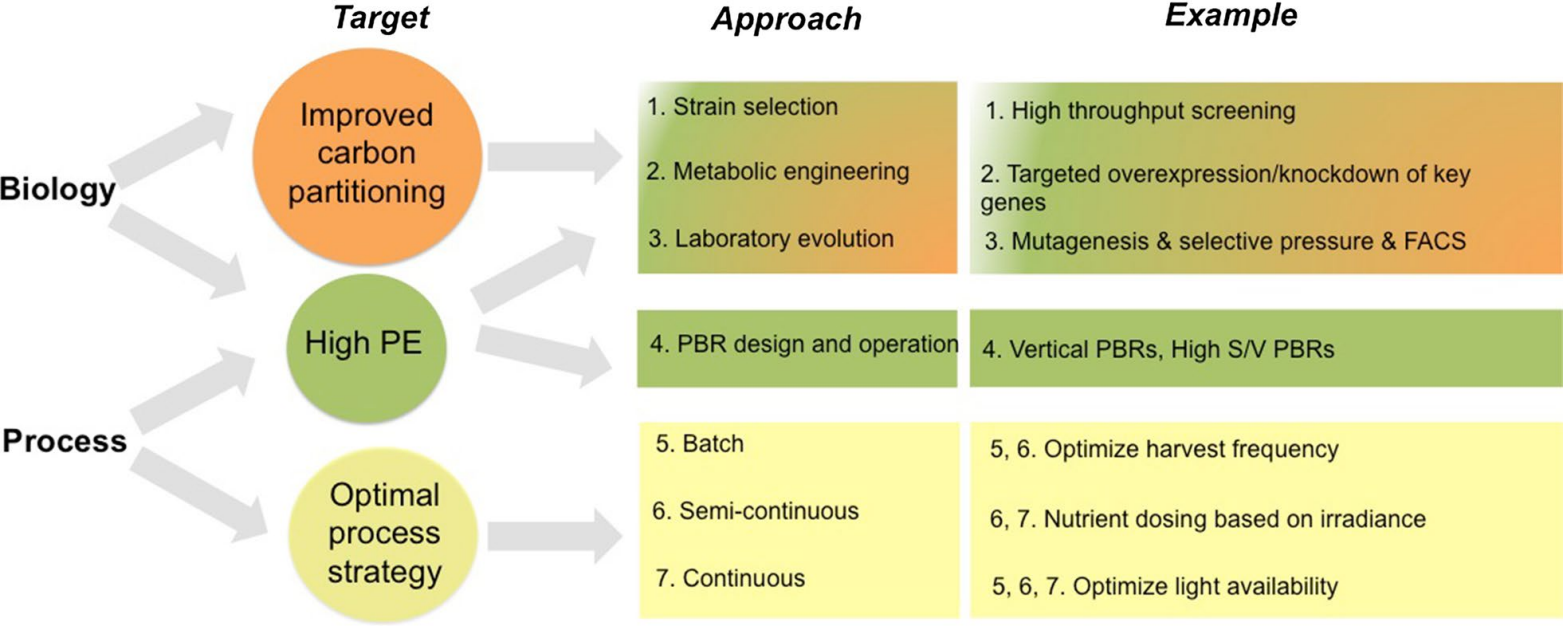
Strategies to increase TAG productivity. *PE* photosynthetic efficiency; *FACS* fluorescence-assisted cell sorting; *S*/*V* surface/volume ratio; *PBR* photobioreactor

Our sensitivity analysis indicates that increasing the TAG productivity is the most influential parameter on cost reduction (Fig. 3). Several approaches for increasing TAG productivity are proposed (Fig. 5). These are essentially related to the selection and/or improvement of the production strain as well as to the optimization of process conditions. In the following sections, the most relevant approaches are discussed and guidelines for improving TAG productivities are presented.

#### Biological approach for higher TAG productivities

##### Exploring the genetic diversity of microalgae

Several screening studies have been performed [24, 29–35]. In practice, only few microalgae (e.g. *Nannochloropsis*, *Chlorella*, *Scenedesmus*, *Neochloris*) have been tested. However, the high genetic diversity of microalgae should be fully explored to select a robust production strain. To reduce risks of contamination, obtain stable productivities and decrease costs during cultivation (e.g. cooling, pH control), microalgae should be isolated from highly selective and extreme environments such as deserts, hot and alkaline-saline waters. Additionally, high-throughput screening protocols (e.g. microfluidics) [33, 36, 37] for which the target product (e.g. TAG) can be quantified [38–40] should be routinely adopted. The predictability of such high-throughput screenings should be validated at lab scale with the selected strains and processes should be developed under simulated outdoor production conditions [34]. Finally, the selected strains should be tested outdoors in pilot PBRs under relevant climate conditions [24, 41, 42].

##### Strain improvement

Besides the natural diversity of microalgae, strain improvement can further enhance TAG productivity. Improving carbon partitioning towards TAG production and photosynthetic efficiency are the two main targets [14, 43, 44]. Recently, successful attempts in increasing TAG productivity and/or content have been achieved either by targeted knockdown of a key gene involved in TAG catabolism [45], or by disabling competitive carbon pathways with starchless mutants [23, 46, 47]. Similar results could also be achieved by decreasing the fraction of other biomass components made during N-starvation (e.g. reducing the carbon flow towards polysaccharide and glycoprotein matrix of the cell wall). Besides metabolic engineering, also adaptive laboratory evolution to a selective pressure [48] combined with FACS (Fluorescence Activated Cell Sorting) [38, 49, 50] or RACS (Raman Activated Cell Sorting) [51] can lead to increased TAG productivities.

Higher photosynthetic efficiencies could be achieved by increasing the electron flow through the electron transport chain [52], the activity and specificity of limiting enzymes, e.g. RuBisCo, involved in anabolic pathways [53, 54] and also with reduced antenna size mutants [55, 56].

#### Process approach for higher TAG productivities

##### Optimization of reactor design

Much research on developing or improving PBR design is ongoing (e.g. optimal distance between panels/loops, culture depth, mixing times, light distribution in the reactor) [57]. The ideal PBR should intercept all available sunlight while ensuring high photosynthetic efficiencies [58, 59] and thus high TAG productivities. This can possibly be achieved with those flat panel designs that allow tilting the reactor to the incoming light (e.g. GWP-III flat panel, F&M Srl, http://www.femolnine.it).

In general, for high TAG productivities, PBRs with a high surface-to-volume ratio (e.g. Solix Biofuels^**®**^ and Proviron Holding NV flat panels and Třeboň, Czech Republic thin-layer cascades ponds) are preferred as, if properly mixed, they ensure high photosynthetic efficiencies and high volumetric TAG concentrations [60–63]. Finally, the selection of optimal designs should be guided by techno-economic analyses considering both biological productivities and production costs associated with each design [13].

##### Operational strategy: batch vs. (semi-)continuous operations

To identify optimal operational strategies for TAG production, much focus has been addressed on the batch vs. (semi-)continuous debate [64–69]. It was shown that a batch process is the most effective strategy for TAG production [14]. This is because batch cultures start with N-replete cells to which a sudden and large energy imbalance is applied (i.e. N-starvation). These cells have a high initial photosynthetic capacity for both biomass and TAG production. Differently, in (semi-)continuous cultures, cells are continuously exposed to limiting conditions, which lead to a lower overall photosynthetic efficiency and productivity compared to batch processes.

## Conclusions

With our techno-economic analysis of a two-step TAG production process in vertically stacked tubular PBRs, we showed that the production costs of TAG-enriched biomass can be substantially decreased by optimizing both process technology and biological performance. Given that TAG productivity is the most influential parameter on production costs, guidelines for achieving higher TAG productivities are discussed in detail. A great potential relies both on strains with enhanced photosynthetic machinery and carbon partitioning towards TAGs and on PBRs able to intercept all sunlight while ensuring high photosynthetic efficiencies. However, high TAG productivities and contents alone do not directly guarantee economic feasibility of the process, when comparing to the present market value of TAGs. Cost-competitiveness strictly relies on the valorization of the whole biomass components and on cheaper PBR designs (e.g. plastic film flat panels). In particular, further research should focus on the development and commercialization of PBRs where active cooling is avoided and stable operating temperatures are maintained by the water basin in which the reactor is placed.

Concluding, with this work we laid down a solid basis for assessing the economic potential of microalgae TAGs and we identified the crucial bottlenecks and future research that is needed to enable profitable and sustainable microalgal TAG production for the commodity markets.

## Additional file

**Additional file 1.** Model description. A detailed account of the main model features is given
