# Supplementary material for: Towards microalgal triglycerides in the commodity markets
Source: Biotechnol Biofuels. 2017 Jul 17;10:188. doi: 10.1186/s13068-017-0873-2 (PMC5514516; doi:10.1186/s13068-017-0873-2)
Supplement: Supplementary file 1 — Additional file 1. Model description. A detailed account of the main model features is given [file 13068_2017_873_MOESM1_ESM.docx]

**Additional file 1**

**1. Model description**

The techno-economic model originally developed by Ruiz et al. [1] for nitrogen replete biomass production was extended with the TAG production phase. Our evaluation includes the cultivation phase in “growth” and “stress” PBRs and the biomass concentration step to obtain 15% w/w algal slurry as final product. The production costs of TAG-enriched biomass are presented.

In our process, nitrogen (N) replete biomass is produced in continuous (chemostat)-operated PBRs (“growth PBRs”) to ensure a continuous supply of inoculum for the TAG accumulation phase for which multiple batch-operated “stress PBRs” are sequentially inoculated and harvested at maximum time-averaged TAG productivity [2] ensuring a constant daily harvest of TAG-enriched biomass (Fig. A1). Projections were made for a 100 ha-scale plant using vertically stacked tubular PBRs in southern Spain (37˚15’ N 6˚ 56’ W). The process is described in detail in the main text. Here an account of the main model features is given. For further details on equations used to calculate heat flows and power consumption for culture recirculation we refer to [1].

**Figure A1 Schematic view of a two-step TAG production process.** For simplicity, only one growth PBR and six stress PBRs are depicted. The growth PBR is operated in chemostat-mode. The outflow of the growth PBR is free of extracellular nitrogen (i.e. just enough nitrogen was fed to the growth PBR to sustain N-replete growth) and, every day, it fills one stress PBR. In each stress PBR, the TAG-accumulation phase starts immediately after inoculation (time to fill one unit is negligible) and runs in batch-mode for six days. The sequential inoculation and harvesting of all stress PBRs over a period of six days ensures a constant daily harvest of TAG-enriched biomass from the entire stress area.

**2. Model inputs**

Our techno-economic model is based on the following inputs: 1) photosynthetic efficiency, i.e. fraction of total light energy converted into chemical energy during photosynthesis, 2) location and 3) photobioreactor.

2.1 Photosynthetic efficiency

Yearly biomass [3] and TAG [4] productivities were calculated using the photosynthetic efficiencies obtained in the outdoor pilot production systems at the AlgaePARC facility in the Netherlands and the total irradiance in southern Spain [1] (Table A1, Eq. A1).

$Yearly biomass productivity=\frac{Light intensity \cdot Phtosynthetic efficiency}{Dry biomass combustion enthalpy}\cdot\frac{300 operational days}{365 days}$ Eq. A1

As explained in the main text, an average photosynthetic efficiency of 2.17% for biomass production (with 4% TAG w/w) and a daily culture dilution rate of 27% were used during the growth phase [3] yielding a retention time of 3.7 days. For the stress phase, an average photosynthetic efficiency of 1.48% for biomass production (containing 24% TAG w/w) was used [4]. Based on the data obtained at pilot scale, we calculated that under low light conditions in the Netherlands (14 mol m^-2^ d^-1^), total TAG productivity is maximal after nine days in the stress reactor, whereas at high light conditions (36 mol m^-2^ d^-1^), the productivity is maximal after six days. Because southern Spain has longer periods of high light compared to the Netherlands, we chose to always harvest the TAG-enriched biomass after six days in the stress PBRs. This retention time of six days can thus be regarded as a 17% daily dilution of the PBRs in the stress area.

**Table A1** Biological model inputs

| **Growth phase (chemostat-operation; 31 m^2^ reactor ground area)** | | | | | |
| --- | --- | --- | --- | --- | --- |
| **Light intensity**  (mol m^-2^ d^-1^) | **N-replete**  **biomass productivity**  (g m^-2^ d^-1^) | **N-replete**  **biomass yield on light**  (g mol^-1^) | **Cellular TAG content**  (%) | **Photosynthetic efficiency**  (%) | **Combustion enthalpy of dry biomass**  (kJ g^-1^) |
| 14 | 6.8 | 0.49 | 4 | 2.12 | 22.5 |
| 36 | 18.4 | 0.51 | 4 | 2.22 | 22.5 |
| **Stress phase^*^ (batch-operation; 4.4 m^2^ reactor ground area)** | | | | | |
| **Average light intensity**  (mol m^-2^ d^-1^) | **TAG-enriched**  **biomass productivity^*^**  (g m^-2^ d^-1^) | **TAG-enriched biomass yield on light**  (g mol^-1^) | **Cellular TAG content**  (%) | **Photosynthetic efficiency**  (%) | **Combustion enthalpy of dry biomass**  (kJ g^-1^) |
| 14 | 4.97 | 0.36 | 19 | 1.75 | 25.2 |
| 36 | 8.38 | 0.23 | 29 | 1.20 | 27.0 |

* At day 6 of cultivation.

To calculate the N- replete and TAG-enriched biomass productivities as well as biomass concentration and TAG content at the two light intensities, mathematical fit of experimental data vs. PFD were used (R^2^ > 0.95).

2.2 Location

The production facility is located in southern Spain (37°15 ́ N 6°56 ́ W).

The location-specific parameters are:

1. Climatic conditions (<http://www.energy.gov/>; <http://www.windguru.cz>; <http://www.sodais.com/eng/index.html>). The climatologic data are based on average hourly data. The irradiance influences both the productivity and the oxygen production that, together with day length and dilution rate, determine the necessary major equipment. Data on temperature, irradiation, relative humidity, wet bulb temperature, dew point temperature and wind speed are used to estimate the requirements for temperature control of the culture.
2. Energy cost
3. Labor cost
4. Employer’s contribution to labor cost
5. Workweek hour

More details are given in section 3.2.

2.3 Photobioreactor

The production occurs in vertical stacked horizontal tubular photobioreactors [1, 5].

This system is made of rigid borosilicate glass transparent tubes. The tubes are stacked parallel to the ground in a vertical structure. A pump is circulating the culture liquid at a velocity of 0.45 m s^-1^ from the tubes to the degasser at the end of the loop and back to the tubes.

A standard unit is defined as one loop of two tubes in opposite directions. The maximum length of the units is limited by oxygen build up and depends on the maximum photosynthetic rate, which is calculated using the productivity at the maximum hourly irradiation (kg biomass m^-3^ h^-1^) and the photosynthetic quotient for the urea (1.11 mol O_2_ mol assimilated CO_2_^-1^), the irradiation, the dissolved oxygen concentration before the degasser and the flow velocity.

Maximum dissolved oxygen concentration before the degasser is set to 300% of oxygen saturation to prevent oxygen inhibition [6] . The gas exchange unit can be connected to several standard units. The volumetric gas-liquid mass transfer coefficient in the degassers is 0.08 s^-1^ for 1.52 volume of air per degasser volume and time. For further details on photobioreactor design we refer to [1] and [5].

*Temperature control*

Maximum culture temperature is = 30°C. When culture temperature is above the setpoint, cooling is activated. Temperature control is performed by a combination of heat exchangers, pumps and cooling water from the sea. Cooling water comes from a 200 m depth at a temperature as given by the National Centers for Environmental Information, National Oceanic and Atmospheric Administration (www.nodc.noaa.gov). The cooling water is pumped through the in-the-culture-submerged heat exchangers. The heat flow in the photobioreactors and the expected temperature of the culture are calculated on an hourly basis. Heat flows are calculated according to [1] considering irradiance, radiation and convection and assuming that the light falling on the ground surface is fully absorbed. The energy consumed by the pumps determines the energy consumption for temperature control. As explained by Ruiz et al. [1], pumps with a shaft power calculated assuming 3 m of water column pressure, 1,027 kg m^-3^ as seawater density and a pump efficiency of 75% are used. The cost of chemical cooling water treatment is 0.004 € m^-3^.

*Power requirement for liquid circulation*

The power consumption to maintain the flow in the tubes is calculated as described by [1] using 1,027 kg m^-3^ as seawater density and a pump efficiency of 75%. Energy losses both due to friction (calculated using the Darcy Weisbach and the Swamee-Jain equations) and bends are accounted in the total energy consumption.

**3. Biomass cost**

The biomass cost (€·kg^-1^) is calculated by dividing the total annual costs (i.e. CAPEX + OPEX) by the total dry biomass annual production. The total annual costs are calculated by summing the annual capital expenses (CAPEX) and the annual operating expenses (OPEX).

3.1 Capital expenses or CAPEX

CAPEX is based on the capital investment, its depreciation and interest. Major equipment (ME, Table A2) is not location-specific and is depreciated over 15 years with 8% interest rate.

**Table A2** Major equipment

| **# Scheme** | **Equipment** | **Stage** | **Capacity** | **Units** | **Cost (€)** | **Power (kW)** |
| --- | --- | --- | --- | --- | --- | --- |
| 1 and 10 | Seawater pump | Growth | 200 | m^3^ h^-1^ | 13,544 | 5.9 |
| 2 | Sterilization | Growth | 59.9 | m^3^ h^-1^ | 117,979 | - |
| 3 | Mixing unit | Growth | 1 | m^3^ | 199,000 | 0.5 |
| 4 | Recirculation pump | Growth and stress | 700 | m^3^ h^-1^ | 28,105 | “Power requirement for liquid circulation” in [1] |
| 5 | Blower | Growth and stress | 200 | m^3^ h^-1^ | 3,027 | 0.99 |
| 5 | Degasser | Growth and stress | 0.66 | m^3^ | 1,214 | - |
| 6 | CO_2_ Supply unit | Growth and stress | 1 | Ha | 4,717 | Insignificant |
| 7 | Pump (cooling) | Growth and stress | 28000 | m^3^ h^-1^ | 595,600 | “Temperature control” in [1] |
| 8 | Piping (cooling) | Growth and stress | 1 m | - | 350 | - |
| 9 | Heat exchanger | Growth and stress | “Temperature control” in [1] | | | |
| 11 | Centrifuge | Stress | 65 | m^3^ h^-1^ | 300,000 | 55 |
| - | Steel structures (poles) | Growth and stress | 3.8 | kg m^-1^ | 621 € ton^-1^ | - |

As explained by Ruiz et al. [1], Lang factors are used to estimate CAPEX: by multiplying MEC by Lang factors the weight of the different items is obtained (Table A3) and, when the cost of a component is not from the current year, the price is updated using the Harmonized Indices of Consumer Prices.

The number of units of ME for each case is based on mass balances for the peak capacity (i.e. for the month with the highest irradiation). For a conservative estimate, the number of processing units is calculated considering operation at 90% of the maximum capacity of equipment and the number of units needed is rounded to the next larger integer. Purchase tax is neglected as it is recoverable, while land is rented at a not location-specific cost of 1.100 € ha^-1^ yr^-1^ (based on price of rented agricultural land in The Netherlands).

**Table A3** CAPEX and OPEX estimating procedure

| **FIXED CAPITAL INVESTMENT** | Direct Cost  (DC) | Major Equipment | MEC |
| --- | --- | --- | --- |
|  |  | Installation costs | 20% MEC |
|  |  | Instrumentation and control | 150% MEC |
|  |  | Piping | 20% MEC |
|  |  | Insulation | 0% MEC |
|  |  | Electrical | 10% MEC |
|  |  | Buildings | 23% MEC |
|  |  | Land improvement | 12% MEC |
|  |  | Service facilities | 20% MEC |
|  | Indirect Cost  (IC) | Construction expenses | 10% DC |
|  |  | Engineering and supervision | 30% MEC |
|  | Other Cost  (OC) | Contractor’s fee | 5% DC |
|  |  | Contingency  (Major equipment) | 15% (DC + IC) |
|  | Working capital | | OPEX first three months of operation |
| **CAPEX** | Depreciation | | (DC + IC + OC)/15 years |
|  | Interest | | 8% depreciation |
|  | Property tax | | 1% depreciation + interest |
|  | Insurance | | 0.6% depreciation + interest |
|  | Purchase tax | | - |
|  | Land | | 1.100 € Ha^-1^ yr^-1^ |
| **OPEX** | Energy | | Calculated from ME consumption |
|  | Labor | | Salaries + Employer’s contribution + Supervision |
|  | Raw materials | | Calculated from mass balances |
|  | Consumables | | Unit cost x #units / lifetime |
|  | Others | Maintenance | 4% MEC |
|  |  | Operating supplies | 0.4% (Electricity + Raw materials) |
|  |  | Contingencies | 15% Raw materials |
|  |  | Overheads | 55% (Labor + Maintenance) |

Table adapted from [1].

3.2 Operational expenses or OPEX

OPEX is the annual sum of:

a) Raw materials

- Nutrients: nutrient concentrations and cost are calculated based on the biomass concentration and composition.
  - CO_2_ amount is calculated from the productivity considering a CO_2_ : biomass ratio of 1.87 derived from the elemental composition of the biomass; C_106_H_181_O_45_N_16_P_17_). In the base case, commercial CO_2_ is used, whereas for the sensitivity analysis the use of flue gas is evaluated.
  - Nitrogen supplied as urea
  - Phosphorous supplied as triple-superphosphate
- Cleaning

Cleaning is performed with 3% of a solution composed of 35% H_2_O_2_ and 7.5% glycerin and small plastic granulates at a concentration of 0.5 Kg·m^-3^. The latter are recovered afterwards and can be reused for three years. These granulates are also added into the broth during cultivation to prevent biofilm formation.

b) Consumables

Glass tubes of the photobioreactor and filtration membranes, the latter used for the optimization scenarios, are considered as consumables. The annual cost of consumables is obtained by multiplying the unit cost by the number of units, and then divided by the lifetime. Tubes have a 20 years lifetime and they are disposed at no cost, whereas filtration membranes have a a three years lifetime.

**Table A4** Raw materials and consumables

|  | | **Price** | **Lifetime (Years)** |
| --- | --- | --- | --- |
| **RAW MATERIALS** | Commercial CO_2_ ^1^ | 184 € ton^-1^ | - |
|  | CO_2_ from flue gas ^1, 2^ | 29 € ton^-1^ | - |
|  | Nitrogen from urea ^1^ | 633 € ton^-1^ | - |
|  | Phosphorus from triple superphosphate ^1^ | 1,155 € ton^-1^ | - |
|  | Chemical cleaning ^3^ | 668 € m^-3^ | - |
|  | Plastic granulate ^4^ | 22.9 € kg^-1^ | 3 |
| **CONSUMABLES** | Glass tubes ^5^ | 4.13 € m^-1^ | 20 |
|  | Microfiltration membranes | 26 € m^-2^ | 3 |

^1^ Necessary amount is calculated from the biomass productivity, an elemental biomass composition C_106_H_181_O_45_N_16_P. A CO_2_: biomass ratio of 1.87 was considered.

^2^ The price accounts for all the upstream operations to concentrate CO_2_ from the flue gas and make it usable in the process [7] as well transportation cost [8]. Transportation cost is calculated conservatively, using 180 km as supply distance; the largest from [8]. For transportation in pipelines, 13.68 kWh ton^-1^ of CO_2_ are necessary, which, together with an energy cost of 0.122 € kWh^-1^ (for Southern Spain; Eurostat, 2014), will determine the final transportation cost for flue gas.

^3^ 3% of a solution composed of 35% H_2_O_2_ and 7.5% glycerin

^4^ Used at a concentration of 0.5 Kg·m^-3^

^5^ 20% installation cost is included

Table adapted from [1].

c) Energy

The energy cost is estimated as the product of the total power consumption and the electricity supply cost in southern Spain (0.122 € kWh^-1^; European Commission. Eurostat, 2014).

d) Labor

The labor cost is calculated from the number of workers (assuming a standard workweek of 40 hours), qualification and cost of working hour (from salary and number of hours per workweek) (Table A1). The employer's contribution is also considered to cover liability of work-related accidents and occupational illness.

**Table A5** Labor cost in southern Spain

| **Minimum wages** ^1^  (€ Yr^-1^) | 9,080 ^1, 2^ |
| --- | --- |
| **Employer's contributions**  (% labor cost) | 23.6 ^3^ |
| **Standard workweek**  (hours) | 40^1^ |
| **Times the minimum wage** | Manager: 6.7  Supervisor: 4.3  Operator: 3 |
| **Supervision** | 20% extra on labor cost |

^1^ Source: European Commission. Eurostat, 2014

^2^ Source: Ministerio de Empleo y Seguridad Social

^3^ Source: Bureau of Labor, Occupational Employment Statistics, 2014, assuming the occupation titles “Industrial production managers”, “First-Line Supervisors of Mechanics, Installers, and Repairers” and “Installation, Maintenance, and Repair Occupations” from the study for plant manager, supervisor and operator, respectively.

e) Maintenance, operating supplies and general plant overheads are calculated as factors of the major equipment, whereas contingencies are calculated as a percentage of raw materials (Table A3).
